# Features of Immunosenescence in Women Newly Diagnosed With Breast Cancer

**DOI:** 10.3389/fimmu.2018.01651

**Published:** 2018-07-16

**Authors:** Lauren Trintinaglia, Lucas Poitevin Bandinelli, Rodrigo Grassi-Oliveira, Laura Esteves Petersen, Marcelo Anzolin, Bruna Luz Correa, Jaqueline Bohrer Schuch, Moisés Evandro Bauer

**Affiliations:** ^1^Laboratory of Immunosenescence, School of Sciences, Pontifícia Universidade Católica do Rio Grande do Sul (PUCRS), Porto Alegre, Brazil; ^2^Graduate Program in Biomedical Gerontology, Pontifícia Universidade Católica do Rio Grande do Sul (PUCRS), Porto Alegre, Brazil; ^3^Developmental Cognitive Neuroscience Laboratory (DCNL), School of Health Sciences, Porto Alegre, Brazil; ^4^Centro Universitário Ritter dos Reis, Porto Alegre, Brazil; ^5^Labvitrus Laboratory, Porto Alegre, Brazil; ^6^National Institute of Science and Technology on Neuroimmunomodulation (INCT-NIM), Rio de Janeiro, Brazil

**Keywords:** childhood maltreatment, breast cancer, immunosenescence, cytomegalovirus, T lymphocytes

## Abstract

Adults exposed to childhood maltreatment have increased stress reactivity. This profile is associated with dysregulation of the immune system, including enhanced inflammatory reactions and accelerated senescence. Subjects exposed to ear stress have increased risk for several age-related diseases, including cardiovascular disease, type II diabetes, and cancer. Although previous studies have reported immune changes in advanced cancer, very little information is available regarding early stage breast cancer. Here, 29 patients with breast cancer were recruited: 15 with history of childhood maltreatment (CM+) and 14 without history (CM−). Twenty-seven healthy women without CM were selected as the control group. Peripheral blood was collected and lymphocyte subsets phenotyped by multi-color flow cytometry (B cells, CD4+ T, CD8+ T, natural killer cells, activated T cells, regulatory T cells, and senescence-associated T cells). Because human cytomegalovirus (CMV) was associated with signatures of early senescence, the CMV serology was determined by ELISA. None of the subjects had IgM reactivity to CMV, excluding acute viral infection. There was a higher proportion of patients with increased CMV IgG levels in the CM+ group as compared to CM− or controls. Different stages of T-cell differentiation can be determined based on the cell-surface expression of the costimulatory molecules CD27 and CD28: ear (CD27+CD28+), intermediate-differentiated (CD27−CD28+), and late-differentiated or senescent T cells (CD27−CD28−). After adjusting for age and education, ear T cells (CD27+CD28+) were found reduced in CM+ and CM− patients (*p* < 0.0001). In contrast, intermediate-differentiated T cells (CD27−CD28+; *p* < 0.0001), senescent T cells (CD27−CD28−; *p* < 0.0001), and exhausted T cells (CD8+CD27−CD28−PD1+; *p* < 0.0001) were found expanded in both CM+ and CM− groups. Our data suggest that features of immunosenescence are associated with newly diagnosed breast cancer, regardless of the CM history.

## Introduction

Childhood maltreatment, such as abuse and neglect, increases the vulnerability for the development of psychiatric disorders and cancer in adult life (1, 2). A large meta-analysis revealed that having multiple adverse childhood experiences was associated with increased risk (OR = 2.3) for development of cancer (3). Adults with history of early life stress have increased reactivity of the stress system, with altered cortisol responses to psychosocial stressors (4, 5). A new cancer diagnosis is associated with important psychological burden and can be understood as a “second allostatic hit” (6), further activating the stress system (7). This profile is also associated with dysregulation of the immune system, mainly characterized by increased levels of proinflammatory cytokines and early senescence (8–11). Therefore, the understanding of biological changes associated with early life stress at the cancer onset is of paramount importance. The monitoring of these biomarkers would be beneficial for planning therapies and optimizing the timing of treatment.

Recent evidence indicated that chronic stress in adults as well as childhood maltreatment may lead to accelerated aging of the immune system (immunosenescence) (12). This was demonstrated by shortened telomeres, and changes in specific lymphocyte populations, including the expansion of T cells with late differentiated profile, and increased senescence and exhaustion cellular markers (e.g., PD1 and KLRG1, respectively) (13, 14). Of note, different stages of T-cell differentiation can be determined based on the cell-surface expression of the costimulatory molecules CD27 and CD28 (15, 16). Previous studies have defined naïve T cells or early differentiated (CD27+CD28+), intermediate-differentiated (CD27−CD28+), and late-differentiated or senescent cells (CD27−CD28−). Furthermore, previous studies suggested that childhood maltreatment was associated with increased serology to cytomegalovirus (CMV) (17), as similarly reported in aging studies (12, 18). The immunosenescence profile is largely unknown in patients with breast cancer, and it is speculated these changes may interfere in the prognosis and treatment response.

In this study, we investigated the presence of immunosenescence markers (lymphocyte subtypes and CMV serology) in women newly diagnosed with breast cancer with and without history of childhood maltreatment.

## Materials and Methods

### Subjects

Twenty-nine women with early breast cancer stage (stages 0–3A) were recruited before starting treatment (chemotherapy, radiotherapy, or surgery) from the Mastology Unit at São Lucas Hospital, PUCRS (Porto Alegre, Brazil). Patients with history of major depression, inflammatory or immune-based diseases, as well as a prior history of breast cancer or presence of others cancers were excluded. Clinical characteristics were assessed through hospital medical records and/or questionnaires. In addition, 36 women without breast cancer were selected as the control group—nine of them were excluded due to history of childhood abuse or neglect.

Childhood maltreatment was investigated by the Childhood Trauma Questionnaire—Portuguese version (19). CTQ is a retrospective 28-item self-report instrument that assesses exposure to sexual, physical and emotional abuse, and physical and emotional neglect (20). In this study, the group with childhood maltreatment (CM+) consisted of participants who reported at least one moderate or severe type of childhood abuse or neglect. The group without childhood maltreatment (CM−) consisted of participants who reported none or low scores of CM. Among the patients, 15 were selected as CM+ and 14 were included in the CM− group. The healthy controls had no history of abuse or neglect. Cases and controls were evaluated for depressive symptoms using the Beck Depression Inventory—Portuguese version (BDI-II) (21). The study protocol was approved by both scientific and ethics committees of PUCRS (Porto Alegre, Brazil) and written informed consent was obtained from all participants.

### Isolation of Peripheral Blood Mononuclear Cells (PBMCs)

Peripheral blood (10 mL) was collected between 10 and 12 h from each participant by venipuncture in EDTA tubes. Plasma was isolated and stored at −80°C. PBMCs were isolate by Ficoll density gradient centrifugation (Ge Healthcare Life Sciences—Marlborough, MA, USA), 30 min at 900 *g*. Cells were counted using a microscope (100×) and viability always exceeded 95%, as judged by Trypan Blue exclusion (Sigma-Aldrich—St. Louis, MO, USA).

### Immunophenotyping

A comprehensive panel of lymphocyte subsets was identified by multicolor flow cytometry. Briefly, PBMCs were washed in flow cytometry buffer (PBS containing 1% FCS and 0.01% sodium azide) and treated with Fc Block solution for 20 min. Cells were stained for 30 min at 4°C with combinations of monoclonal antibodies: anti-CD3 FITC (T cells), anti-CD4 PECy5, FITC and APC (Th cells), anti-CD8 PECy5 (Tc cells), anti-CD19 APC (B cells), anti-CD56 APC [natural killer (NK) cells], anti-CD57 FITC (NK), anti-CD28 APC, anti-CD27 PE, anti-CD69 FITC (early activated cells), anti-CD25 FITC (early activated cells), anti-CD103 FITC (regulatory T cell marker), anti-NKG2 (senescent marker), anti-KLRG1 (senescent marker), and anti-PD1 (exhaustion marker). The differentiation stages of T cells were studied as the following criteria: CD27+CD28+ (early differentiated), CD27−CD28+ (intermediate-differentiated), and CD27−CD28− (senescent cells).

All antibodies were purchased from BD Biosciences (San Jose, CA, USA), except anti-NKG2 (Bio-Techne, Minneapolis, MN, USA), anti-KLRG1 (Biolegend, San Diego, CA, USA), and anti-PD1 (Biolegend, San Diego, CA, USA). After staining, cells were washed, resuspended, and analyzed by flow cytometry. At least 20,000 lymphocytes were identified by size (FSC) and granularity (SSC) and acquired using a FACS Canto II flow cytometer (BD Biosciences). The instrument was checked for sensitivity and overall acquisition. Data were analyzed using Flowjo 7.2.5 software (Tree Star Inc., Ashland, OR, USA).

### CMV Serology

Plasma samples were analyzed for both IgM and IgG antibodies anti-CMV using enzyme-linked immunosorbent assays (ELISAs) (IBL International, Hamburg, Germany). Sensitivity and specificity were estimated to be more than 95%. The optical densities (570/620 nm) were estimated in an ELISA plate reader. Samples were considered positive (reactive) for antibodies anti-CMV when values were above the cut-off of 22 IU/mL for IgG. The detection limit is 0.4 IU/mL for both IgM and IgG anti-CMV. The results are expressed in International units per milliliter.

### Statistical Analyses

All variables were tested for normality of distribution by Shapiro–Wilk tests. For continuous variables, differences between groups (CM+, CM−, and controls) were evaluated by Analysis of Variance (ANOVA) or Kruskal–Wallis (K–W). For categorical variables, differences between groups were compared using chi-square (*X*^2^) test. Generalized Linear Modeling (GzLM) was also used to compare differences between groups adjusting for potential confounders (age and education). Linear or gamma distribution was selected based on the distribution outcome and robust estimation with unstructured working correlation matrix was set. Bonferroni *post hoc* test was used to compare means between groups and adjust the observed significance level considering multiple contrast being tested. Relationship between continuous variables were analyzed by Pearson or Spearman’s correlation tests. Statistical analyses were performed using the Statistical Package for Social Sciences, SPSS Statistics V.20 software (SPSS Inc., Chicago, IL, USA). The significance level was set at α = 0.05 (two tailed).

## Results

### Sociodemographic and Clinical Characteristics

Demographic and clinical characteristics of the samples are summarized in Table 1. All groups were similar regarding BDI scores. Individuals of CM+ and CM− groups differed from control group by age, years of education, and income (all *p* < 0.05). CM+ and CM− groups present similar cancer stage and family history of breast cancer. As expected, higher CTQ scores were observed in the CM+ group compared to CM− and control groups (K–W = 28.4, *p* < 0.001).

**Table 1:** Demographic and clinical data of studied groups.

	Controls (*n* = 27)	CM+ (*n* = 15)	CM− (*n* = 14)	Statistics	Pairwise comparison
Age (years)	40.3 ± 10.3^a^	50.4 ± 9.9^b^	49.4 ± 11.8^c^	*F* = 5.9, *p* = 0.005	b and c > a
Education (years)	18.0 ± 4.0^a^	9.9 ± 4.31^b^	13.7 ± 5.13^c^	*F* = 15.2, *p* < 0.001	b and c < a
Income, monthly (US$)	2,364.6 ± 357.7^a^	686.4 ± 100.1^b^	502.9 ± 95.7^c^	K–W = 28.4, *p* < 0.001	b and c < a
Ethnicity (% Caucasian)	26 (96.3)	10 (66.7)	10 (71.4)	*X*^2^ = 6.2, *p* = 0.04	
Family history (yes)	–	5 (35.7)	8 (61.5)	*X*^2^ = 1.8, *p* = 0.40	
Cancer stage					
Stage I	–	7	5		
Stage II	–	2	5	*X*^2^ = 1.8, *p* = 0.40	
Stage III	–	6	4		
BDI-II	6.2 ± 4.0	9.1 ± 5.5	8.3 ± 6.3	*F* = 1.7, *p* = 0.19	
CTQ	29.1 ± 0.8^a^	53.5 ± 3.4^b^	29.9 ± 1.1^c^	K–W = 28.4, *p* < 0.001	a and c < b

*Data shown as mean ± SD*.

*CM+, breast cancer women with history of childhood Maltreatment; CM−, breast cancer women without history of childhood maltreatment; BDI, Beck Depression Inventory; CTQ, Childhood Trauma Questionnaire*.

*F, Analysis of Variance; X^2^, chi-squared; K–W, Kruskal–Wallis*.

### Major Lymphocyte Subsets

**Figure 1:**
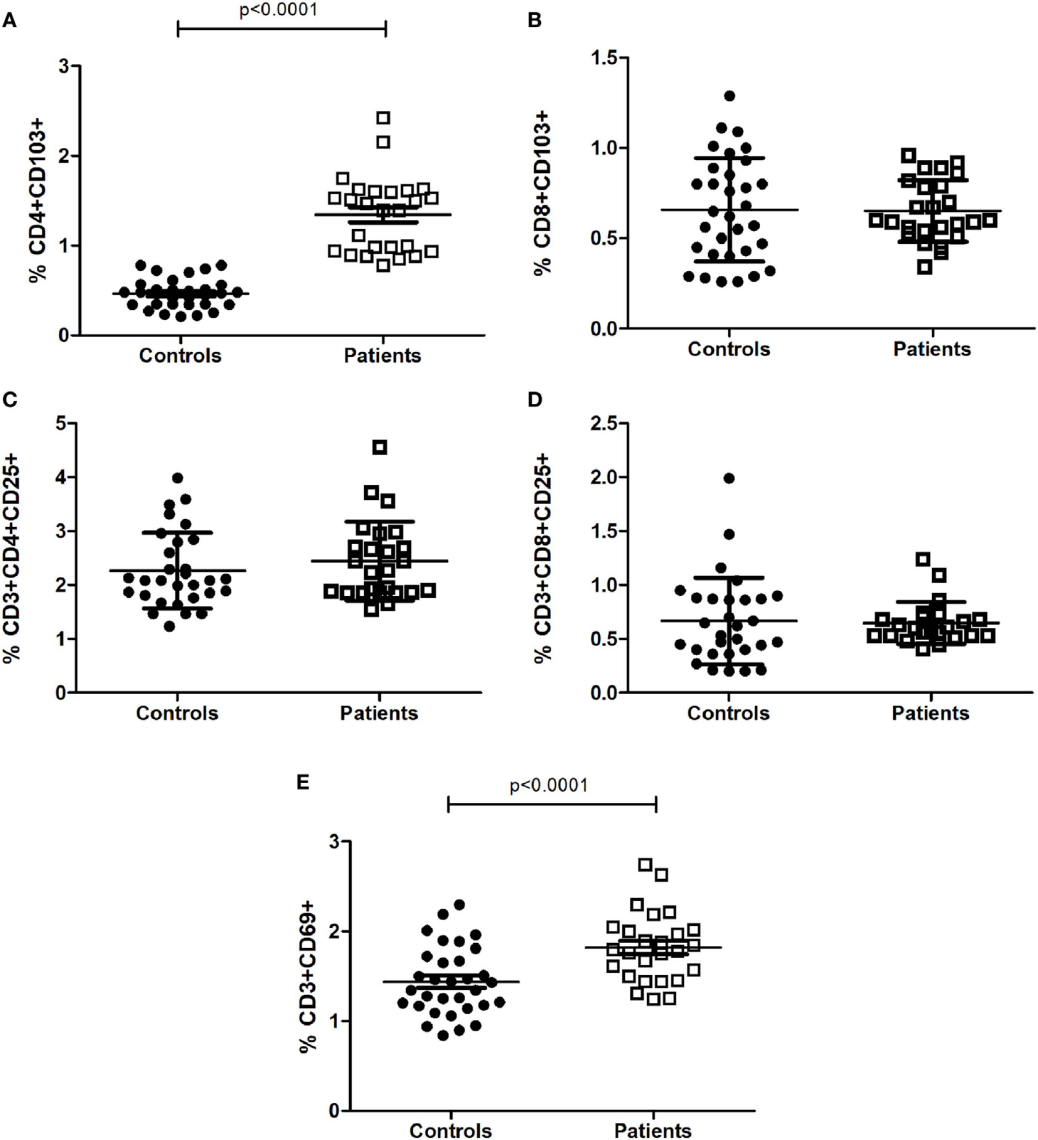
Proportion of T-cell subsets with activated **(C–E)** and regulatory profiles **(A,B)**. Statistical significant differences are indicated. Data were analyzed by Generalized Linear Modeling test (gamma or linear distribution) adjusted for age and years of education.

We investigated different peripheral lymphocyte subpopulations associated with activation and regulatory profiles (Table 2). Activated T cells (CD3+CD69+) and regulatory T cells (CD4+CD103+) were found significantly increased in CM+ and CM− patients (all *p* < 0.0001) compared to controls. Figure 1 shows the mean differences of activation/regulatory markers between patients and controls. Furthermore, the CM+ group had reduced frequencies of CD3−CD19+ B cells (*p* = 0.021) compared to CM− group. Decreased proportions of helper T cells (CD3+CD4+) were observed in the CM− group (*p* < 0.001), but not in the CM+ group or controls. The CD4/CD8 ratio did not differ between groups (*p* = 0.10). No significant differences were found between groups for the remaining subpopulations.

**Table 2:** Immunophenotyping of major lymphocyte subsets.

Markers (%)	Cell type	Controls	CM+	CM−	Statistics (Wald)	*p*-Value
CD3+CD4+	Th	45.4 ± 2.5^a^	41.1 ± 3.7	33.0 ± 2.3^b^	14.2	**<0.001**
CD4/CD8	Ratio	2.2 ± 0.2	2.3 ± 0.4	1.6 ± 0.1	4.5	0.106
CD3+CD8+	Tc	23.7 ± 1.6	21.3 ± 3.2	24.1 ± 2.7	0.4	0.798
CD3−CD19+	B	13.8 ± 0.9	10.0 ± 1.2^a^	15.4 ± 1.8^b^	7.7	**0.021**
CD3−CD56+	NK	8.9 ± 0.8	8.3 ± 1.0	7.1 ± 0.8	2.2	0.327
CD3+CD56+	NK T	6.6 ± 0.7	4.6 ± 0.7	6.2 ± 1.1	2.6	0.271
CD3+CD57+	NK	11.8 ± 1.3	7.2 ± 1.6	10.6 ± 1.6	3.8	0.145
CD3+CD4+CD25+	Activated T cell	2.1 ± 0.1	2.6 ± 0.1	2.4 ± 0.1	2.7	0.251
CD3+CD8+CD25+	Activated T cell	0.6 ± 0.1	0.8 ± 0.2	0.7 ± 0.1	2.1	0.346
CD3+CD69+	Activated T cell	1.3 ± 0.1^a^	1.9 ± 0.1^b^	1.8 ± 0.1^b^	23.3	**<0.0001**
CD4+CD103+	Regulatory T cell	0.4 ± 0.2^a^	1.3 ± 0.1^b^	1.4 ± 0.1^b^	194.8	**<0.0001**
CD8+CD103+	Regulatory T cell	0.7 ± 0.1	0.7 ± 0.2	0.6 ± 0.2	0.8	0.663

*Data shown as mean percentage ± SE. Data were analyzed by Generalized Linear Modeling test (gamma or linear distribution) adjusted for age and years of education*.

*Statistical significant differences are highlighted in bold font*.

*^a,b^Differences between groups (Bonferroni *post hoc* test)*.

*CM+, breast cancer women with history of childhood maltreatment; CM−, breast cancer women without history of childhood maltreatment; Th, helper T cell; Tc, cytotoxic T cell; NK, natural killer cell*.

### Lymphocyte Subsets With Senescence Profile

**Figure 2:**
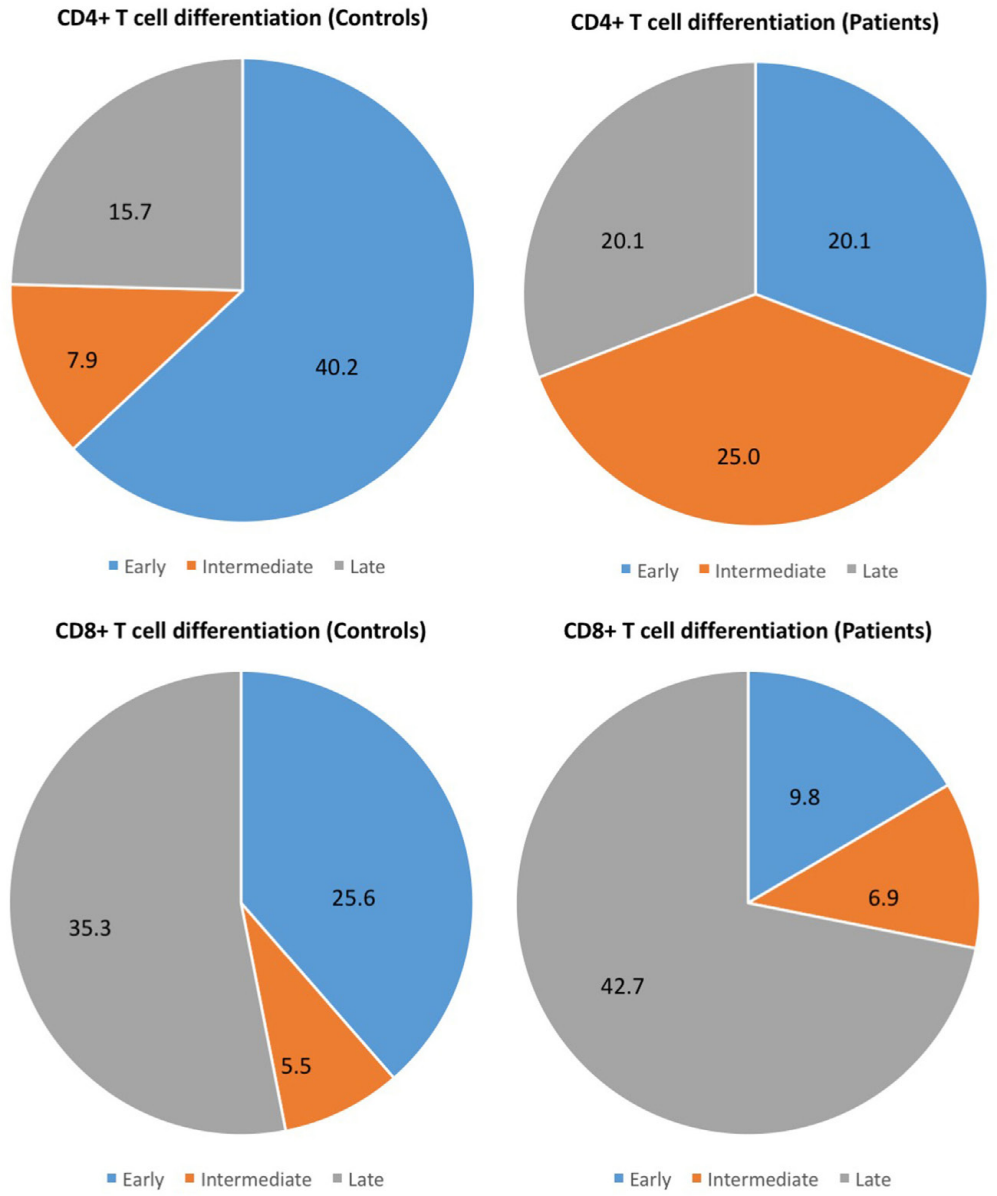
Different stages of T-cell differentiation between patients and controls. The following stages of differentiation can be described based on the expression of cell-surface co-stimulatory molecules CD27 and CD28 (15, 16): early differentiated (CD27+CD28+), intermediate-differentiated (CD27−CD28+) and late-differentiated (CD27−CD28−) T cells. The figure shows the shrinkage of the early differentiated T-cell pool in contrast to the expansion of the pool of late-differentiated T cells in patients.

Different stages of T-cell differentiation can be described based on the expression of cell-surface co-stimulatory molecules CD27 and CD28 (15, 16) (Table 3). The early differentiated T cells (CD4+CD27+CD28+ and CD8+CD27+CD28+) were found significantly reduced in CM+ and CM− patients (all *p* < 0.0001) compared to controls. In contrast, the intermediate-differentiated T cells (CD4+CD27−CD28+), and late-differentiated (senescent) T cells (CD4+CD27−CD28−) were found expanded in patients (all *p* < 0.0001). The Figure 2 shows the shrinkage of the early differentiated T-cell pool in contrast to the expansion of the pool of late-differentiated T cells in patients.

**Table 3:** Different stages of T-cell differentiation and senescence-related markers.

Markers (%)	Cell type	Controls	CM+	CM−	Statistics (Wald)	*p*-Value
CD4+CD27+CD28+	Early differentiated T cell	42.0 ± 4.6^a^	17.5 ± 3.9^b^	19.2 ± 3.9^b^	17.6	**<0.0001**
CD8+CD27+CD28+	Early differentiated T cell	27.9 ± 3.3^a^	8.7 ± 1.2^b^	12.0 ± 2.9^b^	38.1	**<0.0001**
CD4+CD27−CD28+	Intermediate-differentiated T cell	8.74 ± 1.1^a^	25.0 ± 8.8^b^	25.7 ± 8.2^b^	12.9	**0.002**
CD8+CD27−CD28+	Intermediate-differentiated T cell	7.1 ± 0.9	6.9 ± 2.1	8.0 ± 1.8	0.2	0.880
CD4+CD27−CD28−	Late-differentiated T cell	13.9 ± 1.2^a^	21.8 ± 2.1^b^	22.9 ± 1.5^b^	19.8	**<0.0001**
CD8+CD27−CD28−	Late-differentiated T cell	39.4 ± 3.4	34.4 ± 4.6	42.8 ± 5.5	1.6	0.431
CD3+CD56+NKG2+	Senescent NK T cell	0.5 ± 0.1	0.6 ± 0.1	0.7 ± 0.3	3.4	0.179
CD3+CD4+KLRG1+	Senescent T cell	5.1 ± 0.8	10.7 ± 3.0	11.5 ± 4.0	3.4	0.183
CD3+CD8+KLRG1+	Senescent T cell	8.7 ± 0.9	6.1 ± 0.9^a^	9.3 ± 1.5^b^	8.7	**0.013**
CD4+KLRG1+NKG2+	Senescent T cell	5.96 ± 2.8	19.0 ± 4.0	21.0 ± 4.5	5.4	0.065
CD8+KLRG1+NKG2+	Senescent T cell	6.0 ± 0.8^a^	20.0 ± 3.9^b^	24.9 ± 7.7^b^	17.1	**<0.0001**
CD8+CD27−CD28−PD1+	Exhausted T cell	7.6 ± 2.5^a^	78.3 ± 0.10.0^b^	97.8 ± 9.7^b^	55.5	**<0.0001**

*Data shown as mean percentage ± SE. Data were analyzed by Generalized Linear Modeling test (gamma or linear distribution) adjusted for age and years of education*.

*Statistical significant differences are highlighted in bold font*.

*^a,b^Differences between groups (Bonferroni *post hoc* test)*.

*CM+, breast cancer women with history of childhood maltreatment; CM−, breast cancer women without history of childhood maltreatment; NK, natural killer cell*.

Similarly, the senescent T cell CD8+KLRG1+NKG2+ was found expanded in both CM+ and CM− patients (*p* < 0.0001). Also, the early senescent T cell CD3+CD8+KLRG1+ was found significantly increased in the CM− but not in CM+ group or controls (*p* = 0.002). The CD8+CD27−CD28−PD1+ T cells (exhausted T cells) were found expanded in both CM+ and CM− groups when compared to controls (*p* < 0.0001).

### Expression Levels of Cell-Surface Markers

Next, we investigated the expression levels of the studied cell-surface markers by the analysis of the mean fluorescence intensity (MFI), an estimation of the receptor density (Table 4). Of note, we assessed the following biomarkers: CD3, CD19, CD69, CD103, CD27, CD28, KLRG1, NKG2, and PD-1. The CM+ group had higher expression of CD103 in CD4 T cells (*p* < 0.0001) and of NKG2 in CD8 T cells (*p* < 0.0001) as compared to CM− or control groups. Furthermore, the CD27 expression in CD4 T cells was found higher in controls compared to CM+ and CM− groups (*p* = 0.002). In CD8 T cells, higher CD27 and CD28 expression was observed in controls compared to women with breast cancer (*p* = 0.001 and *p* < 0.0001, respectively).

**Table 4:** Expression of activated and regulatory markers as determined by the mean fluorescence intensity (MFI).

Markers	Controls	CM+	CM−	Statistics (Wald)	*p*-Value
**CD3+**					
CD19	4,894 ± 645	4,439 ± 871	4,128 ± 948	0.47	0.790
CD69	358 ± 33	487 ± 78	423 ± 65	3.01	0.222
**CD4+**					
CD103	384 ± 33^a^	854 ± 139^a,b^	568 ± 95^b^	19.92	**<0.0001**
CD27	4,024 ± 244^a,b^	3,088 ± 200^a^	2,869 ± 286^b^	12.84	**0.002**
CD28	3,109 ± 316	2,638 ± 351	2,171 ± 281	4.78	0.091
**CD8+**					
KLRG1	2,638 ± 237	2,964 ± 383	2,897 ± 95	0.95	0.954
NKG2	1,746 ± 334^a^	3,893 ± 344^a,b^	1,603 ± 377^b^	26.9	**<0.0001**
PD-1	2,667 ± 123	2,876 ± 154	2,956 ± 165	1.67	0.756
CD27	3,965 ± 223^a,b^	2,978 ± 265^a^	2,804 ± 290^b^	13.6	**0.001**
CD28	2,543 ± 215^a,b^	1,217 ± 118^a^	1,489 ± 177^b^	25.3	**<0.0001**

*Data shown as mean MFI ± SE. Data were analyzed by Generalized Linear Modeling (linear or gamma distribution) adjusted for age and education years*.

*Statistical significant differences are highlighted in bold font*.

*^a,b^Differences between groups (Bonferroni *post hoc* test)*.

### Influence of CMV on Antibody Titers

**Figure 3:**
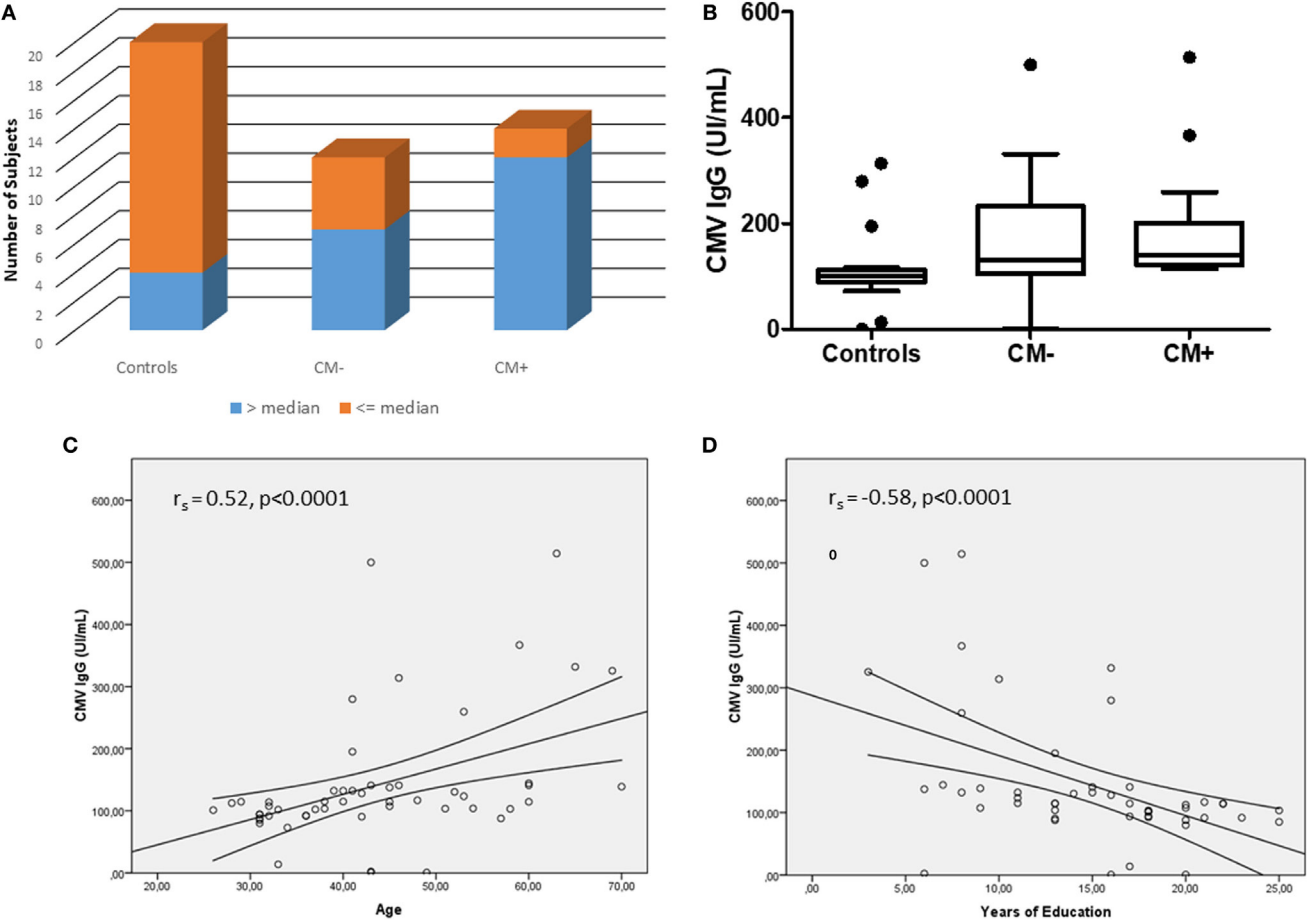
The cytomegalovirus (CMV) serology between studied groups. **(A)** Shows the number of subjects with IgG anti-CMV levels > or ≤median (114.40). **(B)** Shows the CMV IgG levels between groups. After adjusting for age and years of education, no differences were observed between groups (Wald = 0.27, *p* = 0.87). **(C,D)** Show the correlations between CMV IgG serology and age and education, respectively.

The CMV serology was investigated here as another index of accelerated immunosenescence. None of the subjects had IgM reactivity to CMV, excluding acute viral infection. The median IgG anti-CMV titers was 114.40, and there was a higher proportion of subjects with increased serology (>median) in the CM+ group (*n* = 12) as compared to CM− (*n* = 7) and controls (*n* = 4), χ^2^ = 14.67, *p* < 0.001 (Figure 3A). The CM+ patients had increased CMV IgG levels [median = 141.0 (interquartile range; IQR) (124.7–276.0)] as compared to controls [median = 94.1 (85.7–111.2)], *p* < 0.05 (Figure 3B). However, the CM− group had similar CMV IgG levels [median = 126.9 (109.0–137.1)] as compared to CM+ (*p* = 0.22) or control groups (*p* = 1.0). As expected, age (*r*_s_ = 0.52, *p* < 0.0001) and education (*r*_s_ = −0.58, *p* < 0.0001) were correlated to CMV IgG levels (Figures 3C,D). Therefore, when adjusted for these confounders, the CMV IgG levels did not differ between groups (Wald = 0.27, *p* = 0.87).

**Figure 4:**
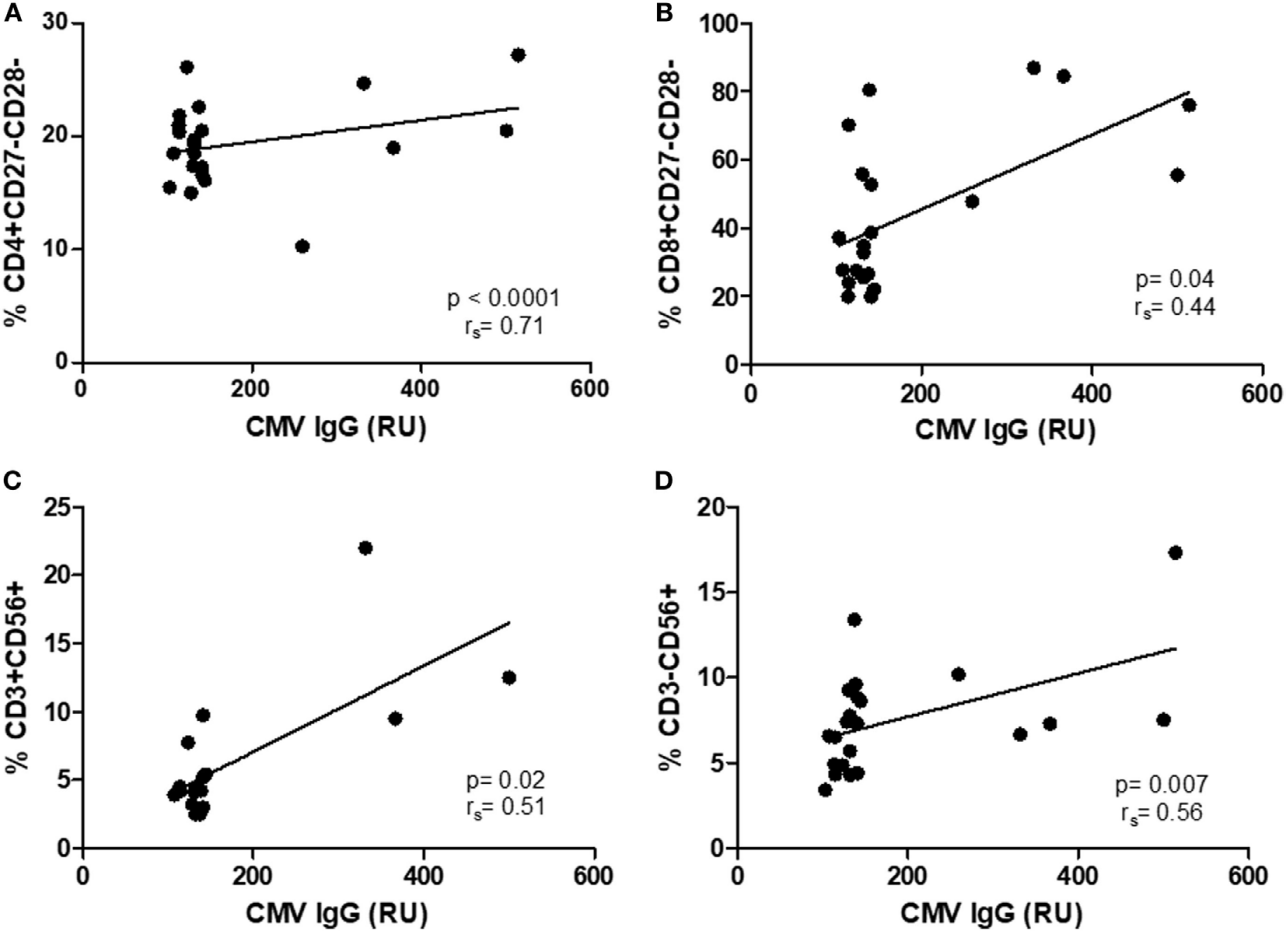
Correlation between IgG anti-cytomegalovirus (CMV) titers and immunosenescence markers in women with breast cancer. **(A–D)** Show the correlations between senescent CD4 and CD8 T cell (*r*_s_ = 0.71, *p* < 0.0001; *r*_s_ = 0.44, *p* = 0.04) and natural killer and NKT (*r*_s_ = 0.51, *p* = < 0.02; *r*_s_ = 56, *p* = 0.007) and anti-CMV titers.

**Figure 5:**
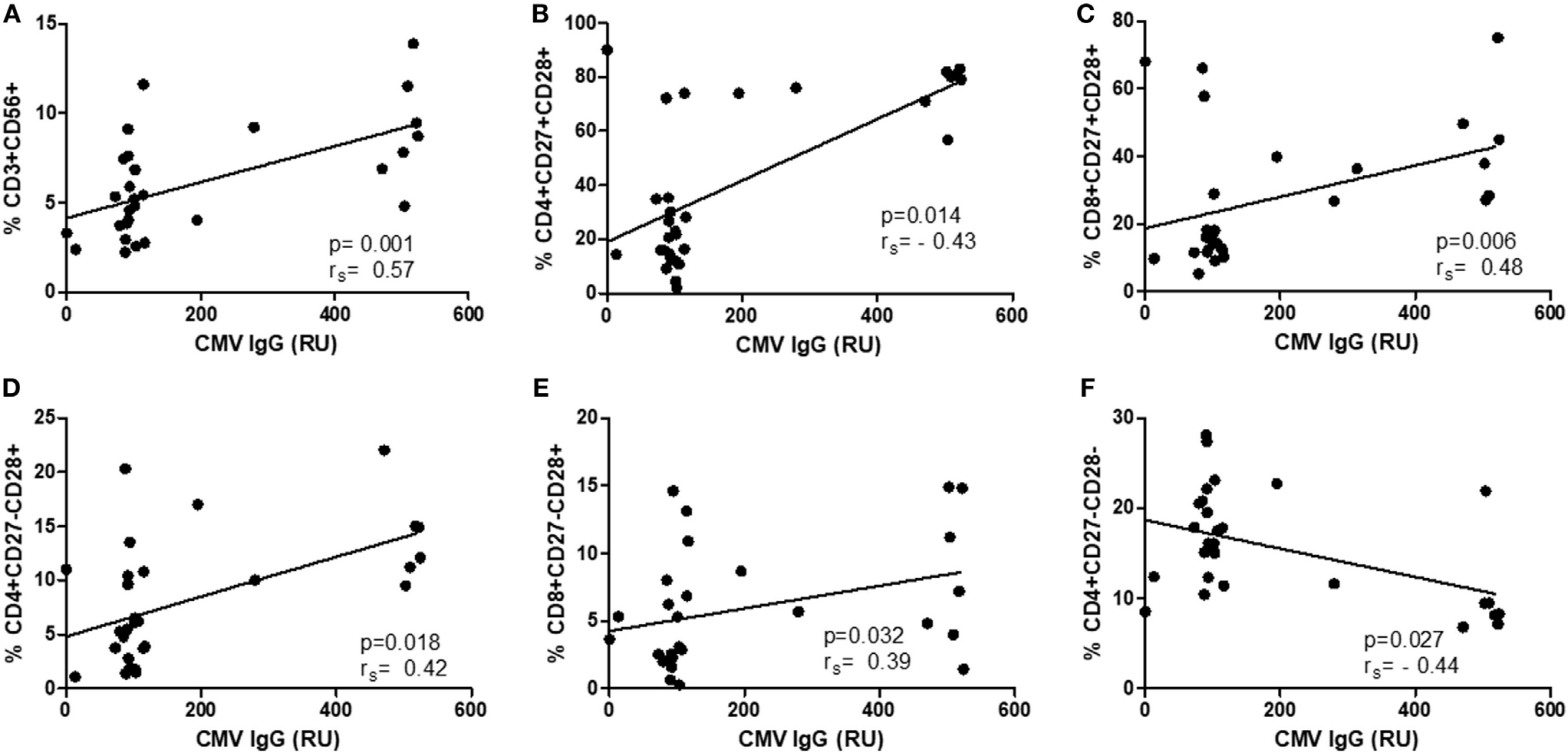
Correlation between IgG anti-cytomegalovirus (CMV) titers and immunosenescence markers in healthy controls. **(A–F)** Show the correlation between CD3+CD56+ (*r*_s_ = 0.57, *p* < 0.001), CD4+CD27+CD28+ (*r*_s_ = 0.43, *p* = 0.01), CD8+CD27+CD28+ (*r*_s_ = 0.49, *p* = 0.06), CD4+CD27−CD28+ (*r*_s_ = 0.42, *p* = 0.01), and CD8+CD27−CD28+ (*r*_s_ = 0.39, *p* = 0.03) and anti CMV–IgG titers.

As there were no differences in CMV IgG levels between CM+ and CM− groups, the following analyses were thus performed without considering childhood maltreatment. The relationships between CMV IgG levels and lymphocyte subsets were analyzed separately in controls and breast cancer patients. In women with breast cancer, positive correlations were found between anti CMV IgG levels and percentage of CD4+CD27−CD28− (*r*_s_ = 0.71, *p* < 0.0001, Figure 4A), CD8+CD27−CD28− (*r*_s_ = 0.44, *p* = 0.04, Figure 4B), NK-T cells (*r*_s_ = 0.51, *p* = 0.02, Figure 4C), and NK cells (*r*_s_ = 0.56, *p* = 0.007, Figure 4D). In the control group, positive correlations were detected between anti CMV IgG levels and the following cell subtypes: CD3+CD56+ (*r*_s_ = 0.57, *p* < 0.001), CD4+CD27+CD28+ (*r*_s_ = 0.43, *p* = 0.01), CD8+CD27+CD28+ (*r*_s_ = 0.49, *p* = 0.06), CD4+CD27−CD28+ (*r*_s_ = 0.42, *p* = 0.01), and CD8+CD27−CD28+ (*r*_s_ = 0.39, *p* = 0.03) (Figure 5). In addition, negative correlations between CD3+CD103+ (*r*_s_ = −0.47, *p* = 0.09) and CD4+CD27−CD28− (*r*_s_ = −0.44, *p* = 0.02) with anti-CMV IgG levels were also observed.

## Discussion

In this study, we observed the presence of a cellular senescence profile in women with breast cancer, regardless of the history of childhood maltreatment. Very little is known about the effects of early life stress on subjects diagnosed with cancer. A prospective study with women with breast cancer explored the effects of childhood adversity on depressive symptoms and immune functions over 37 weeks following breast surgery (22). At initial assessment, women exposed to CM had greater perceived stress, depressive symptoms, as well as lower NK cell activity (NKCA). In that study, clinical features concerning treatment variables (radiation therapy, surgery, time since surgery, use of hormonal treatment), disease severity (ongoing cancer), and CTQ scoring may explain the reported associations between CM and low NKCA. In our study, newly diagnosed patients were recruited (before any treatment) and no differentiation in CTQ scoring could be made because of limited sampling in subscales. The presence of CM, of note, the sexual abuse, has been associated with increased incidence of adulthood cancer (23). Therefore, we speculate that early life stress and disease progression (e.g., treatment) may participate in the “second hit model” (6) to promote synergistically allostatic changes, including those involving the immune system.

Previous studies in the field have described immune changes in breast cancer, usually involving immunotherapeutic aspects and response to treatment. The frequency of activated T-cells is modulated by the presence of tumors, as demonstrated in a study comparing women with breast cancer and healthy controls (22, 24). As observed in our study, there was an increase in the proportion of activated T cells (CD69+) in breast cancer. B cells also seem to be expanded in women with breast cancer (25). We observed a similar increase in the proportion of B cells in the group of women with breast cancer who were not exposed to childhood abuse when compared to healthy controls.

In our study, we observed distinct changes involving aging lymphocytes. Of note, there was an expansion of CD27−CD28− T cells (late-differentiated or senescent T cells) in contrast to reduced proportion of CD28+CD27+ T cells (early differentiated). The presence of the T-cell senescent profile is corroborated by the loss of the CD27 costimulatory molecule, as shown here by reduced CD27 expression, as estimated by the MFI. Similarly, an increase in the proportion of CD8+CD28− T cells was observed in women with breast cancer during chemotherapy (26), and in lung cancer (27). Previous studies have also reported that the increase of this lymphocyte type in cancer patients may be associated with the advanced staging and treatment inefficacy, since some immunotherapeutic interventions require the presence of this costimulatory molecule to be effective. In addition, the expansion of this population of senescent cells can lead to an inefficient antitumor response, due to the decrease of naïve and effector T-cells (26, 28, 29). However, in our study, the staging of the disease was similar between the cases, and it was not possible to evaluate the potential involvement of the lymphocyte subtypes with the clinical outcome.

In their terminal differentiation (i.e., senescence), T-cells also express regulatory markers such as KLRG1 and PD-1 (13). Exposure to chronic stress in childhood can affect the immune system in a similar way to the effects observed during aging (10). Of note, the expression of KLRG1 was found increased in elderly individuals as well as in sexually abused subjects (30, 31). Corroborating this finding, we found an increase in this senescent marker in T-cell subsets of patients, regardless of the history of childhood maltreatment. The PD-1 is another cell-surface marker of late differentiation or exhaustion. The PD-1 and its ligand (PD-L1) have been especially targeted in the area of immunotherapy (checkpoint blockade), given its effectiveness in increasing antitumor response when stimulated by antibodies. The PD-1 is also expressed in most breast tumors (32, 33). In support of our findings, a previous study reported the increase of this marker in patients with early stage breast cancer (34). Furthermore, the PD-1 has been also investigated in chronic viral infections (CMV, HIV, and viral hepatitis). Individuals with these infections had a senescent profile, demonstrated by the increased frequency of PD-1+ expressing T cells (13, 35). Considering the intense relationship between viral infections, cancer, and immunosenescence, we can confirm that this profile of cellular senescence and exhaustion is also present in patients with breast cancer.

The CMV has been associated with features of accelerated immunosenescence, including the expansion of senescent T cells (CD8+CD28−), reduced T-cell repertoire, and increased plasma pro-inflammatory cytokines (IL-6) (12). Furthermore, the presence of acute stressors, such as the diagnosis of cancer, may lead to immune dysregulations implicated with reactivation of latent viruses such as CMV (17). Previously, the presence of fatigue in women with breast cancer undergoing treatment was associated with higher titers of IgG antibodies to CMV (but not EBV) (36). In line, we observed that CM+ group had increased proportion of subjects with higher IgG serology to CMV as compared to CM− or control groups. During aging studies, the CMV infection has been shown to play a role in driving the expansion of late-differentiated T cells (CD28−) (18), and our data corroborate this hypothesis. In particular, the CMV IgG levels were positively correlated with intermediate- or late-differentiated T cells as well as negatively correlated with early differentiated T cells (CD4+CD27+CD28+). We also observed a positive correlation of NK cells with increased CMV IgG titers in women with breast cancer. This result confirms previous data demonstrating that CMV infection can affect innate immunity by expanding the NK cell pool (37). This finding was also observed in individuals without breast cancer (38).

The subgroups of NK cells studied here did not differ between groups, and these data corroborate the finding of Nieto-Velazquez et al. (24). It should be stressed out that the breast cancer patients evaluated here were in early stages, not undergoing treatment. Therefore, the observed results were not influenced by treatment. Witek Janusek et al., on the other hand, have observed that women with breast cancer during treatment (surgery and radiotherapy) with CM history had lower NKCA (22). This divergence can be attributed to the fact that cancer therapy can cause immunomodulatory effects in cellular populations (24, 39).

Most changes concerning the expression of activated and regulatory markers were observed in both groups of patients, regardless of the history of childhood maltreatment. Some MFI changes were found in line with reciprocal increase/decrease in the studied frequency. Indeed, downregulated expression of CD27 and CD28 was observed in PBMCs of patients as compared to controls, in parallel to reduced frequencies of early differentiated T cells (CD27+CD28+) in patients. These two molecules are important co-stimulatory receptors for T cells, and a reduced expression would be associated with poorer cell-mediated immunity. Although the expression of the CD69 and PD1 did not differ between groups, the CM− and CM+ patients had similar expansion in the proportion of early activated T cells (CD3+CD69+) and exhausted T cells (CD8+CD27−CD28−PD1+). It should be noted, however, that cell-surface expression (MFI) is not correlated with the frequency of cell subsets expressing these markers. Neither the CD25 expression nor the frequency of early activated T cells (CD4+CD25+ or CD8+CD25+) differed between groups. However, some alterations were only observed in the CM+ group. Indeed, the expression levels of the regulatory markers NKG2 and CD103 were only found upregulated in T cells of CM+ patients. To gain further understanding, *in vitro* studies are necessary to compare unstimulated and stimulated levels of these markers in PBMCs or isolated T cells.

In addition, future studies should confirm the cellular senescence in breast cancer by investigating additional cellular and molecular markers, not studied here, including the p16/p53 pathway (involved with senescence growth arrest), telomere erosion, epigenetic changes, oxidative stress and pro-inflammatory secretome [senescence-associated secretory phenotype (SASP)] (40). A previous study reported that older patients (>70 years) with breast cancer and colorectal cancer had lower percentages of naïve and recent thymic emigrants of CD8+ T cells, expanded memory T cells, and shorter PBMC telomeres than age-matched controls (41). Senescent cells can be generated by cancer chemotherapy, potentially fueling aspects of disease progression. In a prospective study with type I to III breast cancer, expression of p16INKa were found increased in CD3+ T cells immediately following chemotherapy and remained elevated 1 year after treatment (42). However, telomere erosion was not affected by therapy.

Our data should be interpreted considering some limitations. The sample size of women with breast cancer is relatively small and the cross-sectional design may preclude causal relationships. Future prospective studies are necessary to explore whether the senescence-related immune changes are found previous or consequent of the tumor.

To the best of our knowledge, this is the first study assessing immunological markers in early diagnosis breast cancer with history of CM. Our results suggest that the cellular senescence profile is associated to the diagnosis of breast cancer, regardless of the history of childhood abuse. Longitudinal studies are needed to explore the relationship of this senescent profile with clinical progression and treatment response.

## Ethics Statement

The study protocol was approved by both scientific and ethics committees (No. 48889815.5.0000.5336 3) of PUCRS (Porto Alegre, Brazil) and written informed consent was obtained from all participants.

## Author Contributions

The paper was written by MB, LT, JS, and revised into its final format by all co-authors. Participant recruitment and screening was performed by LT and LB. Flow cytometry panel was setup by LP and LT. Immunophenotyping and cytometric analysis were performed by LP, LT, and MA. Plasma and PBMC isolation were performed by LT and MA. Statistical analysis was performed by LT, JS, and MB. The CMV serology was determined by BLC. The study was conceived by RG-O and MB. All authors read and approved the final manuscript.

## Conflict of Interest Statement

The authors declare that the research was conducted in the absence of any commercial or financial relationships that could be construed as a potential conflict of interest.
